# Nomograms to predict severe PH and survival in COPD patients using non-invasive parameters

**DOI:** 10.3389/fmed.2026.1745477

**Published:** 2026-06-11

**Authors:** Xingxing Sun, Yuan Cao, Hanqing Zhu, Jianhua Xu, Bigyan Pudasaini, Wenlan Yang, Jinming Liu, Jian Guo

**Affiliations:** 1 Department of Pulmonary Function Test, Shanghai Pulmonary Hospital Affiliated to Tongji University, Shanghai, China; 2 Department of Internal Medicine, Columbia Bainuo Clinic, Shanghai, China; 3 Department of Pulmonary Circulation, Shanghai Pulmonary Hospital Affiliated to Tongji University, Shanghai, China

**Keywords:** cardiopulmonary exercise testing, chronic obstructive pulmonary disease, nomogram, severe pulmonary hypertension, survival

## Abstract

**Background:**

This study aimed to develop simplified nomograms for predicting the likelihood of severe pulmonary hypertension (PH) in chronic obstructive pulmonary disease (COPD) patients and for predicting survival in COPD-associated pulmonary hypertension (COPD-PH) patients.

**Methods:**

A total of 179 COPD patients (128 without severe PH, and 51 with severe PH) were analyzed at the Shanghai Pulmonary Hospital Affiliated with Tongji University between 2013 and 2022. Variables, including demographic data and clinical examination findings, were collected. A multivariable logistic regression analysis was used to identify statistically significant PH variables for establishing a nomogram model. The multivariate Cox hazard analysis identified the predictors of death or lung transplantation, which were used to construct a nomogram. The models were evaluated based on discrimination, calibration, and clinical efficacy using the concordance index (C-index), calibration curve, and decision curve analysis.

**Results:**

Peripheral capillary oxygen saturation at peak (peak SpO_2_), peak oxygen consumption per kilogram (peak VO_2_/kg), peak heart rate (peak HR), and pulmonary arterial systolic pressure (PASP) were associated factors for severe PH based on the multivariate logistic regression analysis and were used to develop a nomogram. The C-index for the training and validation cohorts was 0.906 (95% CI: 0.85–0.96) and 0.93 (95%CI: 0.85–1.00), respectively. The areas under the receiver operating characteristic (ROC) curve were 0.906 and 0.93 for the two cohorts. Predictors included in the survival nomogram model were age, diffusing capacity for carbon monoxide percentage of measured to predicted value (DLCO% predicted), and minute ventilation/carbon dioxide output slope (VE/VCO_2_ slope). The model was constructed to predict 1-, 2-, and 3-year survival. The C-index of the nomogram for the training and validation cohorts was 0.80 (95% CI: 0.71–0.89) and 0.69 (95% CI: 0.52–0.86), respectively. The calibration plots were close to the diagonal line in both cohorts. Decision curve analysis (DCA) showed the nomogram model provided a good net benefit.

**Conclusion:**

The nomogram models based on clinical variables from the non-invasive testing offer an individualized tool to predict severe PH in COPD patients and survival in COPD-PH patients.

## Introduction

Pulmonary hypertension (PH) is a well-recognized complication of chronic obstructive pulmonary disease (COPD) and serves as an independent risk factor for adverse outcomes and mortality (1–4). In 2019, the World Symposium on Pulmonary Hypertension (WSPH) defined severe PH in COPD as a mean pulmonary artery pressure (mPAP) of ≥35 mmHg or an mPAP of ≥25 mmHg in the presence of a low cardiac index (CI < 2.0 L/min/m^2^) (5). Although the prevalence of severe PH among patients with COPD is relatively low, ranging from 1 to 4% (6–10), its clinical significance should not be underestimated given the large global burden of COPD (11). Previous studies have suggested that severe PH may represent a distinct pulmonary vascular phenotype (11) that may benefit from earlier identification and targeted therapeutic interventions (5, 12).

Right heart catheterization (RHC) remains the gold standard for PH diagnosis (13). However, its invasive nature, high cost, and limited accessibility in China restrict the widespread clinical application of RHC. Therefore, identifying reliable non-invasive predictors of severe PH would be of substantial clinical value. Although several studies have investigated this issue, the relevant evidence remains limited, and robust predictive tools are still lacking. In addition, the role of non-invasive parameters in predicting mortality among patients with COPD-associated pulmonary hypertension (COPD-PH) has not been fully elucidated.

Therefore, this study aimed to develop nomogram models based on non-invasive clinical parameters to predict the risk of severe PH in patients with COPD and to estimate mortality risk in patients with COPD-PH. These models may facilitate clinical decision-making, improve risk stratification, and support the timely initiation of targeted therapeutic interventions.

### Patient selection

The retrospective study included 179 COPD patients—37 patients without PH, 91 with mild to moderate PH, and 51 with severe PH—who were referred to Shanghai Pulmonary Hospital between 2013 and 2022. The inclusion criteria were defined as follows: (1) patients were diagnosed with COPD based on the criteria outlined by the Global Initiative for Chronic Obstructive Lung Disease (GOLD) (14), with persistent airflow limitation confirmed by a post-bronchodilator forced expiratory volume in the first second / forced vital capacity(FEV1/FVC) of <0.70; (2) all patients underwent RHC and were diagnosed with COPD-PH according to established diagnostic criteria for PH (15), defined as an mPAP of >20 mmHg with no alternative plausible causes identified; (3) patients were in a stable clinical condition at the time of evaluation and completed arterial blood gas analysis, pulmonary function testing (PFT), cardiopulmonary exercise testing (CPET), echocardiography, and laboratory blood tests (16–20); and (4) patients had complete clinical data, and all examinations were performed within a clinically acceptable time interval.

The exclusion criteria were as follows: (1) pulmonary hypertension caused by other conditions, including left heart disease, chronic thromboembolic pulmonary hypertension (CTEPH), connective tissue disease, congenital heart disease, and other systemic diseases associated with PH; (2) other chronic lung diseases that could independently cause PH, such as interstitial lung disease, bronchiectasis, pneumoconiosis, or extensive post-tuberculosis lung damage; (3) acute exacerbation of COPD, severe infection, active malignancy, or an unstable clinical condition at the time of evaluation; and (4) incomplete clinical data or inability to complete key examinations, including RHC, PFT, and CPET.

The severity of PH was determined in accordance with the recommendations from the 6th WSPH (5), in which COPD with severe PH was characterized by an mPAP of ≥35 mmHg or an mPAP of ≥25 mmHg in conjunction with a low cardiac index (CI < 2.0 L min^−1^ m^−2^). The study was approved by the Ethics Committee of Shanghai Pulmonary Hospital.

#### RHC

RHC was performed after the completion of other examinations during hospitalization. Measurements, including right atrial pressure (RAP), mPAP, pulmonary arterial wedge pressure (PAWP), CI, cardiac output (CO), and pulmonary vascular resistance (PVR), were recorded.

#### PFT

PFT was performed using the Master Screen Diffusor (Erich Jaeger Inc., Germany) according to standard protocols (18). Measurements, including forced vital capacity (FVC), forced expiratory volume in 1 s (FEV_1_), residual volume (RV), total lung capacity (TLC), and carbon monoxide diffusing capacity (DLCO), were recorded. The results were expressed as percentages of predicted (% predicted). The diffusing capacity was corrected for hemoglobin concentration.

#### CPET

CPET was performed on an electronically braked cycle ergometer with a breath-by-breath system (MasterScreen-CPX; Jaeger, Hoechberg, Germany) according to standard procedures (19). Measurements, including minute ventilation (VE), breathing frequency (BF), heart rate (HR), load, carbon dioxide output (VCO_2_), oxygen uptake (VO_2_), oxygen pulse (VO_2_/HR), and peripheral capillary oxygen saturation (SpO_2_), were recorded. End-tidal PCO_2_ (PetCO_2_) and end-tidal PO_2_ (PetO_2_) were recorded every 10 s. Ventilatory efficiency (VE/VCO_2_ slope) was determined as the linear regression slope of VE and VCO_2_. The lowest VE/VCO_2_ was determined by averaging the lowest consecutive 90-s data points. The oxygen uptake efficiency plateau (OUEP) was defined as the highest 90-s consecutive stretch of VO_2_ (mL/min)/VE (L/min) (21). Oxygen uptake efficiency slope (OUES) was computed using the least-squares regression of oxygen uptake on the logarithm of the minute ventilation according to the following equation: VO_2_ = a*lgVE + *b*, where the constant “*a*” is referred to as the OUES (21). Oxygen uptake efficiency slope to body surface area ratio (OUESI) was the OUES-to-body surface area ratio.

### Echocardiography

A specialist performed the echocardiography and reported findings according to recognized standards (20). Measurements, including pulmonary arterial systolic pressure (PASP), ascending aorta (Ao), pulmonary artery (PA), and tricuspid annular plane systolic excursion (TAPSE), were recorded.

### Follow-up

The information during follow-up was obtained from outpatient clinic visits or via telephone interviews. The primary endpoint of this study was all-cause mortality or lung transplantation.

### Statistical analysis

Statistical analyses were performed using SPSS 21.0, R version 4.2.2, and X-tile software. Continuous variables were expressed as mean ± standard deviation for normally distributed data or median (interquartile range) for non-normally distributed data, while categorical variables were presented as counts and percentages. Comparisons between groups were performed using Student’s *t*-test or the Mann–Whitney *U* test for continuous variables, and the chi-square test for categorical variables. To reduce the risk of overfitting, candidate variables were first selected based on clinical relevance and results of the univariate analysis, and the number of variables included in multivariate models was limited according to the events per variable (EPV) principle.

For the diagnostic model, variables significantly associated with severe PH in the univariate analysis were entered into the multivariate logistic regression model using forward stepwise selection based on the Akaike Information Criterion (AIC) to identify independent predictors. A nomogram was then constructed to estimate the risk of severe PH in COPD patients. Internal validation was performed using bootstrap resampling. Model performance was evaluated using the concordance index (C-index), receiver operating characteristic (ROC) curve, calibration curve, and decision curve analysis (DCA) to assess discrimination, calibration, and clinical usefulness.

For the prognostic model, univariate and multivariate Cox proportional hazards regression analyses were performed to identify independent prognostic factors for survival in patients with COPD-PH. The optimal cutoff values for the VE/VCO_2_ slope were determined using X-tile software, which identifies the most appropriate threshold based on survival differences between groups, and patients were subsequently divided into three risk groups. According to lung function guidelines (22), DLCO% predicted was categorized as mild, moderate, and severe impairment and assigned corresponding risk levels (low, middle, and high). A survival nomogram was then established based on the independent prognostic variables identified in the multivariate Cox analysis. The predictive performance of the model was evaluated using the C-index and calibration curves with bootstrap internal validation. A two-sided *p*-value of <0.05 was considered statistically significant.

## Results

### Characteristics of patients

Our study included a cohort of 179 patients who were categorized into 2 groups based on the results of RHC: the group without severe PH, comprising 128 patients who either had no PH or mild to moderate PH, and the group with severe PH, comprising 51 patients. The patients’ characteristics are summarized in Table 1. Notably, patients with COPD with severe PH were slightly younger than those without severe PH [62.0 (51.0, 68.0) vs. 64.0 (58.0, 71.0) years, *p* = 0.028]. No significant differences were observed in terms of sex, height, weight, body mass index (BMI), or Global Initiative for GOLD stage (all *p* > 0.05). Detailed patient data are presented in Table 1.

**Table 1:** Characteristics for patients without severe PH and with severe PH.

Characteristics	COPD without severe PH (*n* = 128)	COPD with severe PH (*n* = 51)	*p-*value
Sex, Male/Female	96/32	35/16	0.385
Age, years	64.0 (58.0, 71.0)	62.0 (51.0, 68.0)	**0.028**
Height, cm	163.7 (157.2, 170.0)	162.0 (155.0, 169.9)	0.342
Weight, kg	59.5 (50.0, 67.0)	57.6 (51.0, 69.0)	0.869
BMI, kg/m^2^	21.7 (19.6, 25.0)	22.4 (19.3, 24.5)	0.574
GOLD stage of obstruction			0.440
I	5 (3.9%)	4 (7.8%)	
II	25 (19.5%)	7 (13.7%)	
III	54 (42.2%)	18 (35.3%)	
IV	44 (34.4%)	22 (43.2%)	
Pulmonary hemodynamics			
RAP, mmHg	2.9 ± 2.6	4.0 ± 3.5	**0.045**
mPAP, mmHg	24.0 ± 6.1	43.7 ± 10.4	**<0.001**
PAWP, mmHg	8.0 ± 3.6	9.8 ± 4.6	**0.021**
CO, L/min	5.3 (4.5, 6.4)	5.1 (4.4, 5.8)	0.115
CI, L/min/m^2^	3.2 (2.7, 3.9)	3.2 (2.6, 3.6)	0.264
PVR, WU	3.2 ± 1.7	6.8 ± 2.5	**<0.001**
Pulmonary function test			
FVC, % predicted	60.3 (45.7, 78.9)	42.4 (35.4, 69.2)	**0.008**
FEV_1_, % predicted	40.1 ± 18.9	39.2 ± 20.7	0.875
FEV_1_ to FVC ratio, %	49.9 (41.4, 58.3)	57.1 (49.3, 70.4)	**0.001**
RV, % predicted	196.4 (152.2, 253.7)	161.1 (119.3, 236.9)	**0.043**
TLC, % predicted	117.7 (97.7, 138.9)	100.3 (76.1, 121.6)	**0.001**
DLCO, % predicted	56.4 ± 24.6	38.8 ± 16.3	**<0.001**
Laboratory parameters			
NT-proBNP, pg/mL	193.5 (51.6, 762.1)	961.0 (272.4, 2402.0)	**<0.001**
HGB, g/L	137.5 (126.0, 149.0)	144.0 (129.0, 158.0)	0.129
RBC, 10^12/L	4.7 ± 0.7	5.0 ± 0.9	**0.014**
Blood gas analysis			
PCO_2,_ mmHg	44.3 (38.8, 54.1)	48.4 (40.4, 58.9)	0.320
PO_2_, mmHg	74.0 ± 21.6	62.2 ± 19.3	**0.001**
SO_2,_ %	92.5 ± 6.1	87.0 ± 9.4	**<0.001**
Cardiopulmonary exercise testing			
Warm HR	102.3 (89.7, 112.0)	115.7 (102.3, 124.0)	**<0.001**
Warm BF, /min	23.3 (20.1, 28.7)	27.7 (24.0, 32.0)	**0.001**
Warm VO_2_, mL	502.1 (446.4, 561.9)	485.8 (409.3, 531.0)	**0.031**
Warm VCO_2_, mL	445.0 (384.4, 510.0)	423.3 (364.7, 493.3)	0.104
Warm VO_2_/HR, mL	5.1 (4.2, 5.8)	4.2 (3.6, 5.0)	**<0.001**
Warm VE/VO_2_	27.0 (22.0, 30.9)	24.1 (19.0, 29.1)	0.066
Warm VE/VCO_2_	44.0 ± 8.9	49.6 ± 18.4	**0.007**
Peak load, W	44.5 (28.0, 63.3)	36.3 (27.7, 50.7)	**0.027**
Peak load, % predicted	43.0 (30.0, 60.8)	36.0 (21.8, 48.3)	**0.025**
Peak HR	119.2 (106.9, 134.9)	129.3 (118.7, 142.0)	**0.005**
Peak BF, /min	27.7 (24.3, 33.3)	33.0 (29.0, 36.3)	**0.002**
Peak VO_2_, mL	702.5 (573.3, 867.0)	599.3 (443.7, 667.3)	**<0.001**
Peak VO_2_, % predicted	47.8 ± 14.8	37.9 ± 14.3	**<0.001**
Peak VO_2_/Kg, mL/Kg/min	11.9 (10.2, 14.7)	10.3 (8.3, 12.1)	**<0.001**
Peak VCO_2_, mL	678.8 (513.5, 853.2)	545.3 (425.3, 690.0)	**0.001**
Peak O_2_/HR, mL	5.9 (4.7, 7.2)	4.4 (3.6, 5.4)	**<0.001**
Peak SpO_2_, %	86.8 (75.5, 94.2)	75.0 (64.4, 81.3)	**<0.001**
Peak VE/VO_2_	27.9 (22.2, 33.1)	25.2 (17.7, 29.5)	**0.019**
Peak VE/VCO_2_	38.9 (32.5, 45.7)	42.3 (33.9, 55.5)	0.054
OUEP, mL/L	28.7 (23.7, 34.3)	26.3 (21.5, 31.2)	**0.028**
LOWEST VE/VCO_2_	38.0 (32.0, 44.7)	40.5 (34.6, 52.2)	0.073
VE/VCO_2_ slope	28.2 (22.7, 36.4)	30.7 (21.3, 46.8)	0.386
OUES	0.6 (0.4, 0.7)	0.8 (0.5, 1.0)	**0.002**
OUESI	0.3 (0.2, 0.4)	0.5 (0.3, 0.6)	**0.001**
Echocardiography			
PASP, mmHg	46.0 (36.0, 51.8)	67.5 (59.0, 79.0)	**<0.001**
Ao, diameter, cm	2.5 ± 0.4	2.6 ± 0.3	0.212
PA, diameter, cm	2.6 ± 0.4	2.8 ± 0.5	**0.003**
TAPSE, cm	2.0 (1.8, 2.3)	1.9 (1.5, 2.1)	**0.001**
TAPSE/SPAP	0.05 ± 0.01	0.03 ± 0.01	**<0.001**

Values are expressed as mean ± SD, *n* (%), or median (interquartile range) and percentage of measured to predicted values (%pred). BMI, body mass index; GOLD, Global Initiative for Chronic Obstructive Lung Disease; RAP, right atrial pressure; mPAP, mean pulmonary artery pressure; PAWP, pulmonary arterial wedge pressure; CI, cardiac index; CO, cardiac output; PVR, pulmonary vascular resistance; FVC, forced vital capacity; FEV_1_, forced expiratory volume in 1 s; RV, residual volume; TLC, total lung capacity; DLCO, carbon monoxide diffusing capacity; NT-proBNP, N-terminal pro-brain natriuretic peptide; HGB, hemoglobin; RBC, red blood cell; PaCO_2_, arterial carbon dioxide tension; PaO_2_, arterial oxygen tension; SaO_2_, oxygen saturation; HR, heart rate; BF, breathing frequency; VO_2_, oxygen uptake; VCO_2_, rate of carbon dioxide production; VE, minute ventilation; SpO_2_, peripheral capillary oxygen saturation; OUEP, oxygen uptake efficiency plateau; OUES, oxygen uptake efficiency slope; OUESI, oxygen uptake efficiency slope to body surface area ratio; PASP, pulmonary arterial systolic pressure; Ao, ascending aorta; PA, pulmonary artery; TAPSE, tricuspid annular plane systolic excursion. A *p*-value of <0.05 is represented by bold values.

### Hemodynamic and laboratory parameters

Patients with COPD with severe PH exhibited worse RAP, mPAP, PAWP, and PVR compared with those without severe PH, while CO and CI showed no significant differences.

The levels of NT-proBNP were markedly higher in the severe PH group than in the non-severe PH group [961.0 (272.4, 2402.0) vs. 193.5 (51.6, 762.1) pg/mL, *p* = 0.000]. Conversely, red blood cell count and hemoglobin levels were lower in the severe PH group (*p* = 0.014 and *p* = 0.129, respectively). Arterial oxygen tension (PO_2_) and oxygen saturation (SO_2_) differed significantly between the two groups (74.0 ± 21.6 vs. 62.2 ± 19.3 mmHg, 92.5 ± 6.1 vs. 87.0 ± 9.4%, all *p* < 0.05), whereas no significant differences were observed in arterial carbon dioxide tension (PaCO_2_).

### Pulmonary function and exercise parameters

Patients with COPD without severe PH demonstrated significantly higher FVC% predicted, RV% predicted, TLC% predicted, and DLCO% predicted compared with those with severe PH (all *p* < 0.05; Table 1). The FEV_1_/FVC% predicted did not exhibit significant differences.

During the warm-up stage, HR, BF, and VE/VCO_2_ were lower in patients with COPD without severe PH than in those with severe PH (all *p* < 0.05). Conversely, VO_2_, VO_2_/HR, and SpO_2_ were higher in the non-severe PH group. VCO_2_ and VE/VO_2_ showed no significant differences. At the peak stage, load, load% predicted, HR, BF, VO_2_, VO_2_% predicted, VO_2_/Kg, VCO_2_, VO_2_/HR, SpO_2_, and VE/VO_2_ all exhibited significant differences between the two groups (all *p* < 0.05), with the exception of VE/VCO_2_. Parameters reflecting gas exchange efficiency, including OUEP, OUES, and OUESI, also demonstrated statistically significant differences between the two groups (all *p* < 0.05). The lowest VE/VCO_2_ and VE/VCO_2_ slope were similar (*p* > 0.05).

Among the echocardiography parameters, PASP and PA diameter increased, while TAPSE, Ao/PA diameter, and TAPSE/PASP decreased significantly in both COPD patients without severe PH and those with severe PH (all *p* < 0.05). Detailed data are presented in Table 1.

### Identifying risk factors for COPD with severe PH

Multivariate logistic regression analysis was applied to non-invasive clinical parameters associated with severe PH. It showed that reduced peak SpO_2_ (OR = 0.95, *p* = 0.01), peak VO_2_/Kg (OR = 0.72, *p* < 0.001), and elevated values of peak HR (OR = 1.04, *p* = 0.005) and PASP (OR = 1.06, *p* = 0.00) were independent predictors of severe PH in COPD patients. Detailed data are shown in Table 2.

**Table 2:** Multivariable analysis of factors correlated with severe PH.

Variables	OR	CI	*p*-value
Peak SpO_2_, %	0.95	0.91–0.99	0.01
Peak VO_2_/Kg mL/Kg/min	0.72	0.59–0.86	0.00
Peak HR	1.04	1.01–1.06	0.005
PASP, mmHg	1.06	1.03–1.09	0.00

OR, odds ratio; 95% CI, 95% confidence interval. Peak SpO_2_, peripheral capillary oxygen saturation at peak; peak VO_2_/kg, peak oxygen consumption per kilogram; peak HR, peak heart rate; PASP, pulmonary arterial systolic pressure.

### Nomogram for risk factors of severe PH

**Figure 1:**
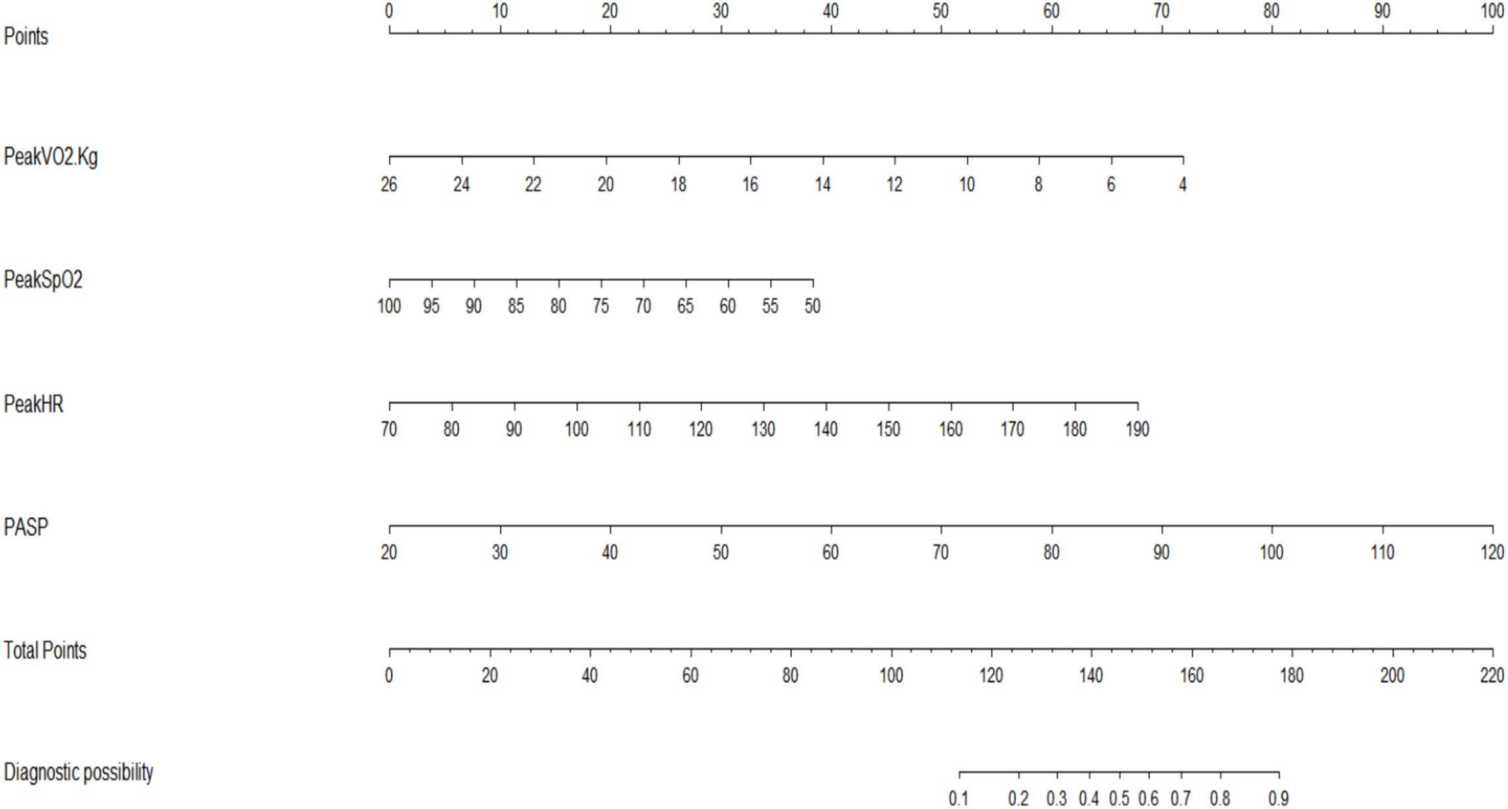
Nomogram for predicting the probability of COPD patients with severe PH. Peak SpO_2_, peripheral capillary oxygen saturation at peak; peak VO_2_/kg, peak oxygen consumption per kilogram; peak HR, peak heart rate; PASP, pulmonary arterial systolic pressure.

A nomogram was constructed using four factors (Figure 1). The score of each risk factor corresponded to the score on the top-scoring axis, and the sum of the scores of each variable was recorded as the total score. The value corresponding to the total score on the bottom axis was the probability of severe PH in COPD patients (Figure 1).

### Prediction model performance

**Figure 2:**
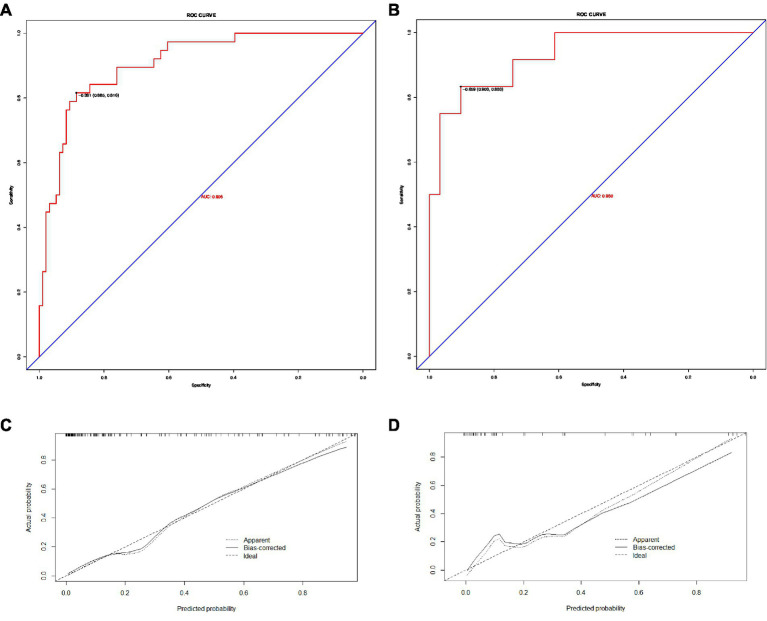
Receiver operating characteristic (ROC) curves and calibration curves for predicting the risk of COPD patients with severe PH. The ROC curves derived from the nomogram demonstrate excellent accuracy in predicting severe PH in COPD patients in both the training cohort **(A)** and the validation cohort **(B)**. Calibration curves of the nomogram for the training cohort **(C)** and for the validation cohort **(D)** are shown.

**Figure 3:**
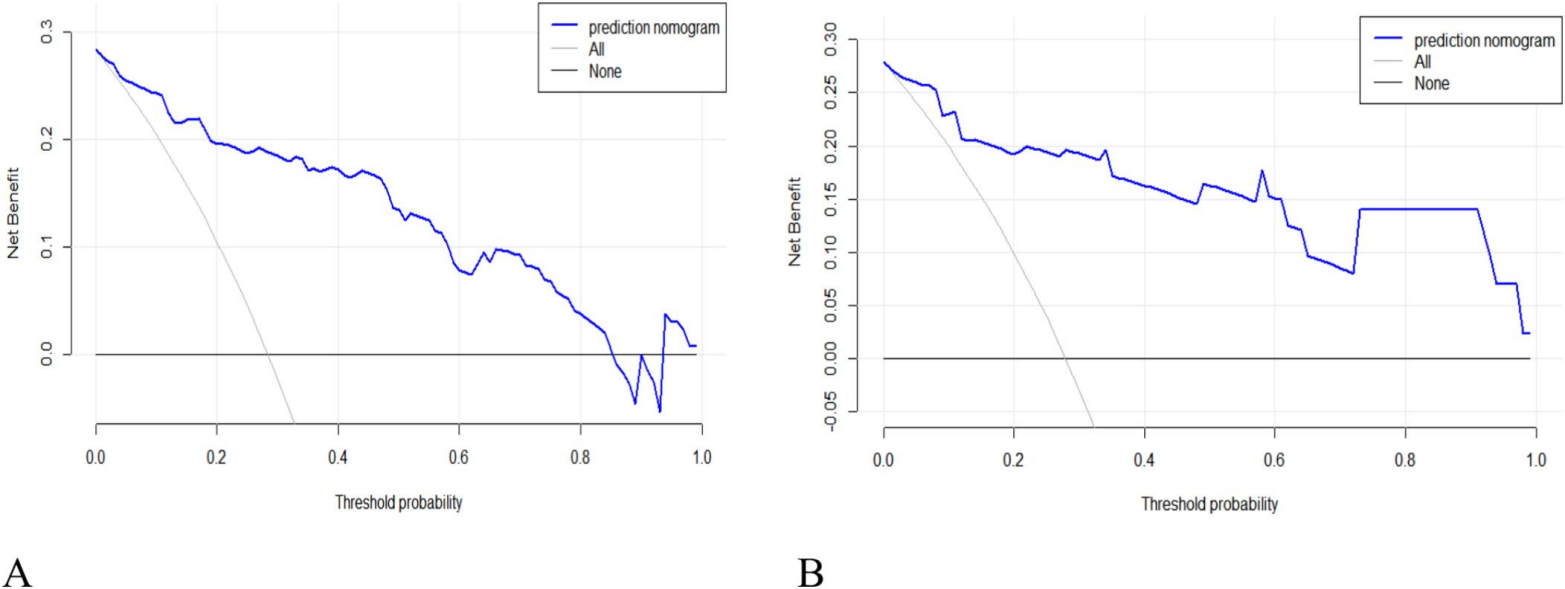
Decision curve analysis using the nomogram model. The *y*-axis measures the net benefit. The blue line represents the decision curve for the clinical model; the black line represents the net benefit when it is assumed that no COPD patients have severe PH; and the grey line represents the net benefit when it is assumed that all COPD patients have severe PH. The preferred strategy is the one with the highest net benefit at any given threshold. **(A)** Decision curve of the training cohort, and **(B)** decision curve of the validation cohort.

The nomogram model achieved modest efficiency, with a concordance index of 0.906 (95% CI: 0.85–0.96) for the training cohort and 0.93 (95% CI: 0.85–1.00) for the validation cohort, respectively. The areas under the ROC curve were 0.906 and 0.93 for the two cohorts, respectively (Figures 2A,B). The calibration curves for the training and validation cohorts were close to the diagonal line (Figures 2C,D). The decision curve analysis for both cohorts showed that the nomogram model provided a greater net clinical benefit (Figures 3A,B).

### Survival

A total of 123 COPD-PH patients were followed up in this study. Patients were randomly divided into a training cohort (*n* = 84) and a validation cohort (*n* = 39) in a 2:1 ratio. A comparison of the baseline characteristics was made between the two cohorts (e-Table 1). The median follow-up time was 2.5 (1.4, 5.0) years and 3.3 (1.6, 4.9) years in the two cohorts, respectively. Overall, 34 patients died, and 1 underwent lung transplantation during follow-up. Overall 1-, 2-, and 3-year survival rates were 94.6, 83.7, and 75.0%, respectively.

### Survival nomogram development

In the univariate analysis, age, FVC (% predicted), FEV_1_ (% predicted), FVC to FEV_1_ ratio, DLCO (% predicted), and ventilatory efficiency parameters (PetO_2_, PetCO2, VO_2_/VE and VE/VCO_2_ during the warm up and peak stage, and OUEP, lowest VE/VCO_2_, and VE/VCO_2_ slopes) were significantly associated with overall survival in the training cohort. Moreover, based on the above parameters, age (OR = 1.06, *p* = 0.002), DLCO (% predicted) (OR = 0.98, *p* = 0.045), and VE/VCO_2_ slope (OR = 1.02, *p* = 0.005) were selected for the multivariate Cox regression analysis (Table 3).

**Table 3:** Univariable and multivariable Cox regression model analyses of overall survival in the training cohort.

Variable	Univariate analysis		Multivariate analysis	
	HR (95% CI)	*p*-value	HR (95% CI)	*p-*value
Age, years	1.06 (1.02, 1.1)	0.001	1.06 (1.02, 1.10)	0.002
Sex	1.01 (0.48, 2.11)	0.986		
BMI, kg/m^2^	0.99 (0.91, 1.08)	0.862		
BSA, m^2^	0.64 (0.10, 4.32)	0.651		
Pulmonary function test				
FVC, % predicted	1.02 (1.00, 1.03)	0.028		
FEV_1_, % predicted	1.02 (1.00, 1.04)	0.027		
FVC to FEV_1_ ratio, %	1.01 (0.99, 1.03)	0.014		
DLCO, % predicted	0.97 (0.95, 0.99)	0.018	0.98 (0.96, 0.99)	0.048
Laboratory parameters				
NT-proBNP, pg/mL	1.00 (1.00, 1.00)	0.001		
Blood gas analysis				
PCO_2_, mmHg	0.99 (0.96, 1.02)	0.542		
PO_2_, mmHg	1.00 (0.99, 1.02)	0.608		
Cardiopulmonary exercise testing				
Warm PETO_2_	1.05 (1.01, 1.09)	0.012		
Warm PETCO_2_	0.95 (0.91, 0.99)	0.012		
Warm RER	9.42 (1.75, 50.62)	0.009		
Warm VO_2_/VE	0.91 (0.85, 0.96)	0.001		
Warm VE/VCO_2_	1.03 (1.01, 1.05)	0.002		
Peak load, W	0.98 (0.97, 0.99)	0.022		
Peak PETO_2_	1.04 (1.01, 1.07)	0.014		
Peak PETCO_2_	0.96 (0.93, 0.99)	0.007		
Peak VO_2_/VE	0.93 (0.89, 0.98)	0.002		
Peak VE/VCO_2_	1.02 (1.01, 1.04)	0.001		
OUEP, mL/L	0.92 (0.87, 0.97)	0.003		
LOWEST VE/VCO_2_	1.04 (1.02, 1.06)	0.000		
VE/VCO_2_ slope	1.02 (1.01, 1.03)	0.000	1.02 (1.01, 1.03)	0.005
Echocardiography				
SPAP, mmHg	1.00 (0.99, 1.02)	0.818		
TAPSE, cm	0.57 (0.19, 1.69)	0.311		

BMI, body mass index; GOLD, Global Initiative for Chronic Obstructive Lung Disease; RAP, right atrial pressure; mPAP, mean pulmonary artery pressure; PAWP, pulmonary arterial wedge pressure; CI, cardiac index; CO, cardiac output; PVR, pulmonary vascular resistance; FVC, forced vital capacity; FEV_1_, forced expiratory volume in 1 s; RV, residual volume; TLC, total lung capacity; DLCO, carbon monoxide diffusing capacity; NT-proBNP, N-terminal pro-brain natriuretic peptide; HGB, hemoglobin; RBC, red blood cell; PaCO_2_, arterial carbon dioxide tension; PaO_2_, arterial oxygen tension; SaO_2_, oxygen saturation; HR, heart rate; BF, breathing frequency; VO_2_, oxygen uptake; VCO_2_, rate of carbon dioxide production; VE, minute ventilation; SpO_2_, peripheral capillary oxygen saturation; OUEP, oxygen uptake efficiency plateau; OUES, oxygen uptake efficiency slope; OUESI, oxygen uptake efficiency slope to body surface area ratio; PASP, pulmonary arterial systolic pressure; Ao, ascending aorta; PA, pulmonary artery; TAPSE, tricuspid annular plane systolic excursion.

**Figure 4:**
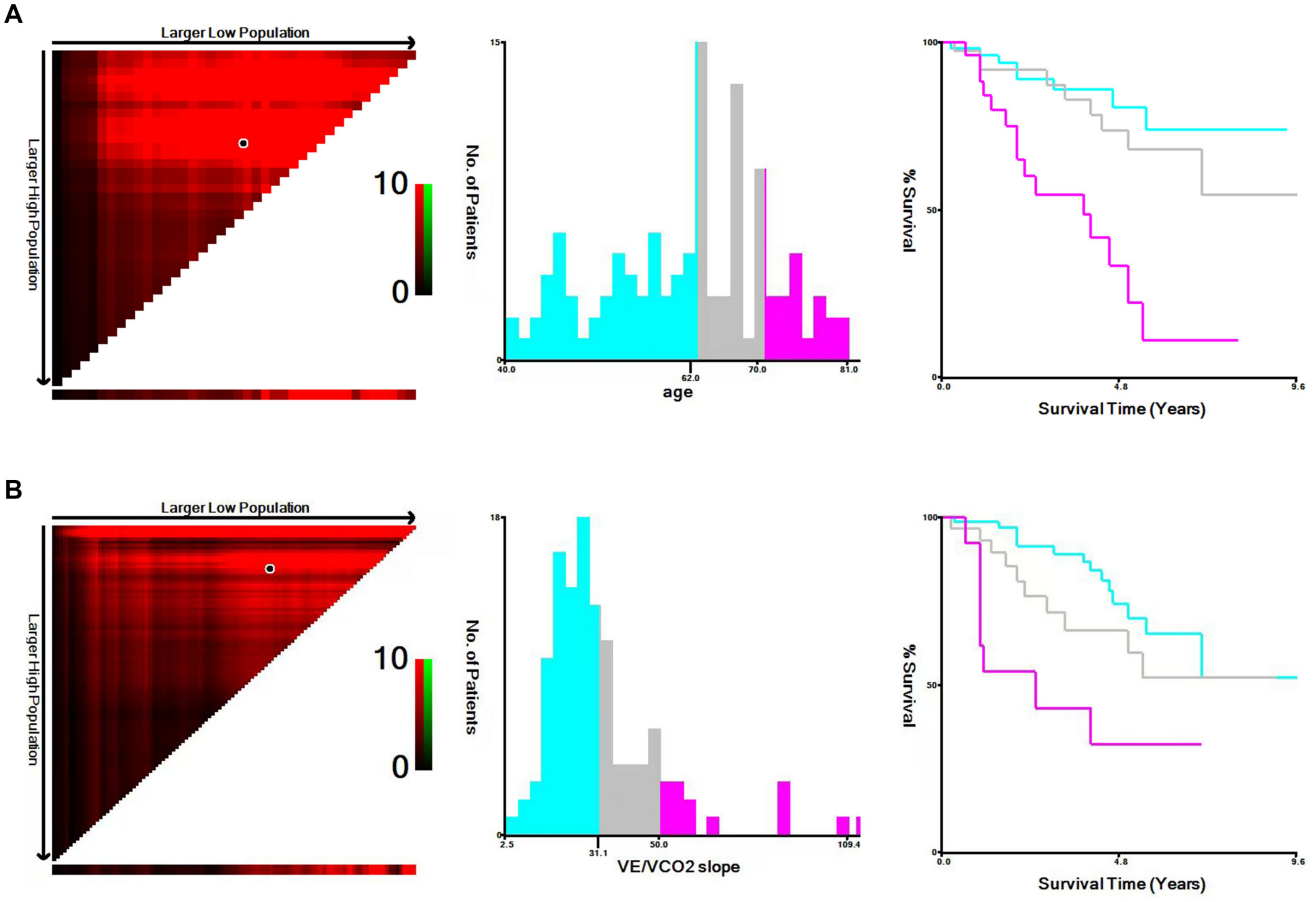
Cutoff values for echocardiographic parameters calculated using the X-tile program. X-tile analyses of age **(A)** and VE/VCO_2_ slope **(B)** levels in the cohort population with COPD-PH. X-tile plots for the cohort patients are shown in the left panels; black circles highlight the cutoff values, which are also shown in histograms (middle panels). The Kaplan–Meier plots are presented in the right panels.

The high-, middle-, and low-risk subgroups were divided according to all-cause mortality using the X-tile program. Then, age and VE/VCO_2_ slope were transformed into ternary variables based on cut-off values determined using the X-tile software (Figure 4).

**Figure 5:**
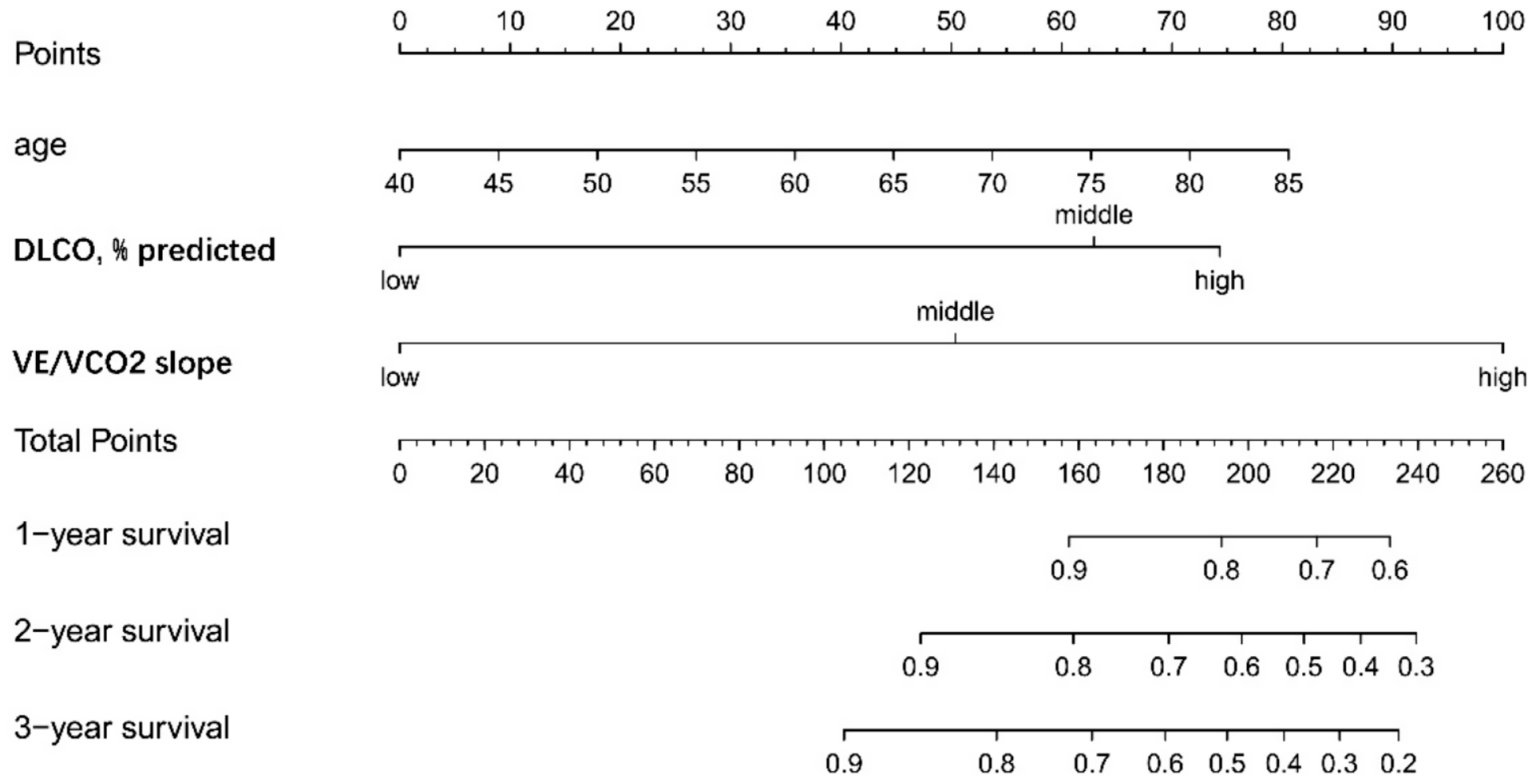
Nomogram for predicting all-cause mortality in COPD-PH. BMI, body mass index; GOLD, Global Initiative for Chronic Obstructive Lung Disease; RAP, right atrial pressure; mPAP, mean pulmonary artery pressure; PAWP, pulmonary arterial wedge pressure; CI, cardiac index; CO, cardiac output; PVR, pulmonary vascular resistance; FVC, forced vital capacity; FEV_1_, forced expiratory volume in 1 s; RV, residual volume; TLC, total lung capacity; DLCO, carbon monoxide diffusing capacity; NT-proBNP, N-terminal pro-brain natriuretic peptide; HGB, hemoglobin; RBC, red blood cell; PaCO_2_, arterial carbon dioxide tension; PaO_2_, arterial oxygen tension; SaO_2_, oxygen saturation; HR, heart rate; BF, breathing frequency; VO_2_, oxygen uptake; VCO_2_, rate of carbon dioxide production; VE, minute ventilation; SpO_2_, peripheral capillary oxygen saturation; OUEP, oxygen uptake efficiency plateau; OUES, oxygen uptake efficiency slope; OUESI, oxygen uptake efficiency slope to body surface area ratio; PASP, pulmonary arterial systolic pressure; Ao, ascending aorta; PA, pulmonary artery; TAPSE, tricuspid annular plane systolic excursion.

A nomogram was constructed using three factors (Figure 5). The score of each risk factor corresponded to the score on the top points axis. The sum of the scores of each variable was recorded as the total score. The value corresponding to the total score on the bottom axis represented the predicted 1-, 2-, and 3-year overall survival probabilities.

### Prediction model performance

**Figure 6:**
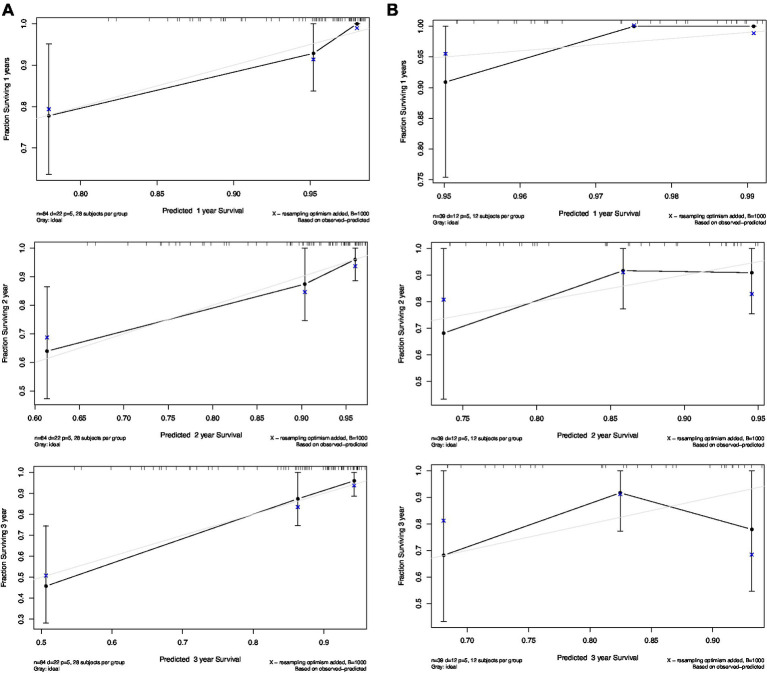
Calibration curves of 1-, 2-, and 3-year survival probability for the training cohort **(A)** and the calibration curve of 1-, 2-, and 3-year survival probability for the validation cohort **(B)**.

The model exhibited good discriminative ability, with a C-index of 0.80 (95% CI: 0.71–0.89) and an internal validation C-index of 0.69 (95% CI: 0.52–0.86). Figure 6, presents calibration plots for 1-, 2- and 3-year survival across the training and validation cohorts. The plots show strong correlation, confirming the high reliability of this nomogram.

## Discussion

This study was conducted to identify severe PH in COPD patients and to predict their survival outcomes. This study performed a comprehensive evaluation using non-invasive clinical parameters to develop individualized predictive models, an approach that remains relatively limited in the existing literature. For the diagnostic model, the variables, including peak SpO2, peak VO_2_/kg, peak HR, and PASP, were integrated to construct a nomogram for predicting severe PH in COPD patients. For the prognostic model, a second nomogram was established based on age, DLCO % predicted, and VE/VCO_2_ slope to estimate survival in COPD-PH patients. The careful development and internal validation of these models highlight their potential clinical utility in risk stratification and individualized management.

The inclusion criteria comprised COPD patients with rigorously confirmed diagnoses, well-characterized disease manifestations, and management within our specialized pulmonary circulation department. The diagnosis of PH was confirmed by RHC, with careful exclusion of other forms of PH, thereby ensuring the accuracy and reliability of the clinical data.

Our findings further confirmed that severe PH in COPD patients is significantly associated with impaired diffusion capacity, elevated NT-proBNP levels, and reduced PaO_2_ and SO_2_, reflecting more extensive pulmonary vascular damage. In addition, CPET parameters highlighted marked impairment in exercise tolerance, circulatory function, and gas exchange efficiency in this patient population (6, 23–25).

Our research has unveiled a nomogram model anchored on peak SpO_2_, peak VO_2_/Kg, peak HR, and PASP, proving to be pivotal in diagnosing severe PH in the COPD population. This severe PH is delineated as a distinct entity, characterized by impaired diffusion and marked exertional dyspnea, owing to a combination of aggravated airway obstruction and pronounced vascular restructuring (6, 11, 26). The restructuring process, catalyzed by accelerated cellular growth and matrix protein accretion, amplifies the clinical manifestations of PH (6). We used CPET, a well-established method for comprehensively assessing physiological responses to exercise (27).Consistent with prior studies, peak VO_2_ played a prominent role in our analysis. Beyond existing evidence, we further demonstrated its value for specifically detecting severe PH in patients with COPD (24, 28–30). COPD patients complicated with PH also presented lower SaO_2_ at peak exercise, which reflected impaired gas exchange. This finding can be explained by multiple mechanisms: increased sympathetic activity, persistent hypoxemia and right ventricular dysfunction. Collectively, these abnormalities lead to a higher HR, a robust indicator of PH severity (24, 26, 28, 31–33). The incorporation of echocardiography and the pivotal role of PASP in PH diagnosis and progression assessment are well established (20, 34, 35). However, the presented nomogram, grounded on these four strategic clinical indicators, transcends existing knowledge, offering a refined diagnostic lens specifically tailored for the identification of severe PH in COPD patients, augmenting the existing body of work with novel insights and applications.

This model represents a significant improvement over existing screening methods. Existing clinical practice often relies on single echocardiographic indicators such as PASP, but their accuracy in COPD patients can be compromised due to emphysema interference. The study model achieves a shift from a single-parameter approach to multidimensional integration by combining CPET parameters (which assesses overall cardiopulmonary functional reserve and oxygenation), cardiac chronotropic function (peak HR), and echocardiographic hemodynamic information (PASP). This integration more comprehensively reflects the characteristic “cardiopulmonary coupling” dysfunction observed in severe PH. Furthermore, by statistically fusing multiple indicators to output individualized prediction probabilities, the model transitions from qualitative or semi-quantitative assessment to a standardized, quantitative risk evaluation. This model enables more sensitive identification of high-risk individuals among COPD patients with atypical clinical presentations or discordant functional test results by capturing declines in functional reserve during exercise. Consequently, this model holds the potential to facilitate earlier intervention. It is important to emphasize that this nomogram serves as an efficient, non-invasive risk stratification tool designed to aid in selecting high-risk patients who require confirmation through right heart catheterization (the gold standard), rather than replacing invasive diagnostic procedures.

Although the models developed in this study demonstrated high predictive performance (such as a C-index >0.90 for the severe PH diagnostic model), this model explicitly emphasizes the exploratory nature of this research. Such high performance of the model should, first and foremost, be regarded as a strong proof of concept, indicating the potential of a carefully selected set of non-invasive parameters to accurately identify the high-risk subgroup of COPD patients with concomitant severe PH. However, this very positioning implies a risk of optimism bias in the model. While the remarkably high C-index undoubtedly highlights the significant potential value of this marker set, it further underscores the critical importance of rigorous external validation in future independent, multicenter, prospective cohorts with larger sample sizes. Only through external validation can the model’s generalizability be accurately assessed, and its true performance level in real-world applications be confirmed.

Our investigation unveiled a predictive model, intricately crafted by integrating age, DLCO % predicted, and VE/VCO_2_ slope, demonstrating proficiency in forecasting all-cause mortality among COPD-PH patients. Utilizing X-tile software, we discerned optimal cut-off values, stratifying age into three distinct categories, each associated with significant differences in survival outcomes. This methodological choice was primarily based on dual considerations of clinical utility and methodological robustness: clear categorical cut-off values provide an intuitive hazard ratio for bedside decision-making; given the limited sample size of this study, this approach avoids the potential misspecification error associated with forcing continuous variables into a linear form within the model, allowing for a more robust capture of potential threshold effects or non-linear relationships between the variables and the outcome. The X-tile software identifies potential “natural” breakpoints in the data by maximizing inter-group survival differences, representing a data-driven yet clinically interpretable strategy. We fully acknowledge the limitations of this tool and therefore regard it as a preliminary exploratory instrument, emphasizing that the categorical variables and cut-off points derived therefrom must undergo rigorous external validation.

Historically, a meticulous cluster analysis of 572 COPD patients, as delineated in the extant literature, underscored not only the pivotal role of lung function but also the intrinsic link between age, associated comorbidities, and patient prognosis (36). Our findings echo and augment this narrative, unveiling DLCO’s nuanced role. A reduced DLCO, as corroborated by preceding studies, emerges as a standalone harbinger of adverse outcomes among patients with PH secondary to chronic lung diseases and is intricately linked to emphysematous changes and pulmonary vascular morphological alterations (37–41, 47, 48). In a seminal study by Schwaiblmair et al. (42), a VE/VCO_2_ slope exceeding 60 delineated a patient cohort beset with an approximately six-fold increase in mortality risk within a 24-month period. This intricate correlation between VE/VCO_2_ slope and key hemodynamic indices was echoed in subsequent studies, fortifying its role in prognostic assessment (43). The innovation lies in the synergistic evaluation of these non-invasive parameters, providing a robust tool for clinicians in the nuanced prognostication of COPD-PH patients. This integrative approach not only aligns with but extends the frontiers of existing knowledge, underscoring the contribution to this evolving discourse.

Previous studies have often centered on echocardiographic and selected non-invasive parameters to predict severe PH and survival in COPD patients (23, 27), yet a comprehensive and systematic analysis remains elusive. Kovacs’ et al. (23) study demonstrated the predictive efficacy of NT-proBNP, PASP, and the PA-to-Ao diameter ratio, derived from chest CT scans, in forecasting severe PH and associated survival outcomes. Our study distinguishes itself through a markedly different patient cohort and methodology. In contrast to Kovacs’ mixed Group 1, 2, 3, and 4 PH categories, our research is specifically tailored to COPD-PH patients. We observed a higher proportion of men and a predominance of patients classified within GOLD stages 3 and 4, indicative of severe obstructive ventilatory dysfunction. This contrasts with Kovacs’ focus on patients within the GOLD 1 and 2 stages.

The observed severity in our study could potentially be attributed to the late stage at which Chinese patients seek medical intervention or the constraints in diagnostic facilities at secondary healthcare centers. Our study incorporates a broader spectrum of CPET parameters, enhancing the depth of cardiopulmonary functional insights. While prior research, such as that by Jiang et al. (44), concentrated on the prognostic implications of isolated clinical tests, our study pioneers the use of comprehensive nomograms integrating multiple non-invasive parameters. These nomograms present a nuanced approach for assessing severe PH and survival in COPD-PH patients, potentially heralding enhanced management protocols for those grappling with concurrent pulmonary vascular disease and obstructive airway conditions.

The research approach of this study aligns with cutting-edge explorations in other medical fields. For instance, in oncology, researchers are dedicated to integrating multi-modal data using artificial intelligence to predict treatment responses, or using non-invasive techniques such as radiomics to replace invasive biopsies for precise prediction of disease phenotypes (45, 46). These studies share the same core vision as ours: to develop high-performance non-invasive predictive models that can identify high-risk patients in a safer and more convenient manner, optimize clinical decision-making processes, and ultimately benefit patients. Drawing on methodological experiences from these fields—such as applying more advanced machine learning algorithms to optimize our parameter combinations or utilizing methods such as restricted cubic splines to more finely characterize the relationship between continuous variables and risk—represents an important direction for future research.

### Study limitations

This study has several limitations. First, it is a retrospective, single-center analysis with a limited sample size and statistical power. Second, some clinical information was incomplete, and imaging data were not collected. Furthermore, many patients did not reach the anaerobic threshold during cardiopulmonary exercise testing, leading to the exclusion of related parameters from the analysis, which may introduce bias. Methodologically, we used the X-tile software to dichotomize continuous variables. While this approach helps identify clinically meaningful cut-off points, the determined thresholds depend on this specific dataset, carry a risk of overfitting, and the categorization process results in the loss of continuous gradient information of the variables. The most important limitation of this study is that the model performance was evaluated only by internal validation and has not been validated in an independent external cohort; therefore, its generalizability remains uncertain. Future research should involve external validation of this nomogram in larger-scale, prospective, multicenter cohorts. More advanced methods, such as restricted cubic splines, could also be considered to more finely characterize the complex relationship between continuous variables and risk, thereby facilitating the translation of the model into clinical practice.

## Conclusion

COPD patients with severe PH exhibit exacerbated ventilatory dysfunction, impaired exercise endurance, and reduced gas exchange efficiency. Our study, characterized by an extensive analysis of numerous non-invasive parameters, culminated in the development of a nomogram incorporating peak SpO_2_, peak VO_2_/Kg, peak HR, and PASP. This predictive tool is designed to offer individualized risk assessment for severe PH in COPD patients. Furthermore, we constructed an additional prognostic model based on age, DLCO % predicted, and VE/VCO_2_ slope, aimed at forecasting survival outcomes in COPD-PH patients. Application of this nomogram could equip clinicians with enhanced predictive capacity, facilitating accurate estimation of 1-, 2-, and 3-year overall survival rates in the COPD-PH cohort. The internal validation results indicate that the model shows promising predictive potential within the present study cohort. Future external validation in larger, multicenter populations is warranted to confirm its generalizability and clinical utility.

## Data Availability

The original contributions presented in the study are included in the article/supplementary material, further inquiries can be directed to the corresponding author.

## Glossary

COPD: chronic obstructive pulmonary disease;
PH: pulmonary hypertension
RHC: right heart catheterization
CPET: cardiopulmonary exercise testing
PFT: pulmonary function testing
BMI: body mass index
GOLD: Global Initiative for Chronic Obstructive Lung Disease
RAP: right atrial pressure
mPAP: mean pulmonary artery pressure
PAWP: pulmonary arterial wedge pressure
CI: cardiac index
CO: cardiac output
PVR: pulmonary vascular resistance
FVC: forced vital capacity
FEV1: forced expiratory volume in 1 s
RV: residual volume
TLC: total lung capacity
DLCO: carbon monoxide diffusing capacity
NT-proBNP: N-terminal pro-brain natriuretic peptide
HGB: hemoglobin
RBC: red blood cell
PaCO_2_: arterial carbon dioxide tension
PaO_2_: arterial oxygen tension
SaO_2_: oxygen saturation
HR: heart rate
BF: breathing frequency
VO_2_: oxygen uptake
VCO_2_: rate of carbon dioxide production
VE: minute ventilation
SpO_2_: peripheral capillary oxygen saturation
OUEP: oxygen uptake efficiency plateau
OUES: oxygen uptake efficiency slope
OUESI: oxygen uptake efficiency slope to body surface area ratio
PASP: pulmonary arterial systolic pressure
Ao: ascending aorta
PA: pulmonary artery
TAPSE: tricuspid annular plane systolic excursion
ROC: receiver operating characteristic curves
AUC: area under the curve
OR: odds ratio
CI: confidence interval
